# Integrating evolutionary signals and predicted protein structure reveals localized molecular divergence in *Cereus* lineages

**DOI:** 10.1007/s10709-026-00293-4

**Published:** 2026-09-25

**Authors:** Danilo T. Amaral, João Alfredo Teodoro, Maria Izadora Oliveira Cardoso, Evandro Marsola Moraes, Fernando Faria Franco, Isabel A. S. Bonatelli

**Affiliations:** 1 Centro de Ciências Naturais e Humanas, Universidade Federal do ABC (UFABC), Avenida dos Estados, 5001, Bloco A, 504-3 Room, Santo André, SP 09210-580 Brazil; 2 Departamento de Biologia, Centro de Ciências Humanas e Biológicas, Universidade Federal de São Carlos (UFSCar), Sorocaba, Brazil; 3 Químicas e Farmacêuticas, Departamento de Ecologia e Biologia Evolutiva, Universidade Federal de São Paulo, Instituto de Ciências Ambientais, Diadema, SP Brazil; 4 Químicas e Farmacêuticas, Programa de Pós-Graduação em Ecologia e Evolução, Universidade Federal de São Paulo, Instituto de Ciências Ambientais, Diadema, SP Brazil

**Keywords:** Adaptation, Positive selection, Stability, Structure, Variability

## Abstract

Understanding how positive selection shapes molecular evolution at the protein level remains a major challenge, particularly in non-model species. Here, we integrated phylogenetic evidence from candidate genes evolving under lineage-specific positive selection with comparative structural and biophysical analyses to investigate patterns of molecular divergence in the *Cereus fernambucensis-C. insularis* clade, which occupies contrasting island and continental environments. Based on a previous branch-site analysis, five orthogroups were selected for downstream analyses. Orthologous protein structures were predicted and compared, variability hotspots were identified, pockets proximity were evaluated, and the stability effects of lineage-specific substitutions were estimated. Across orthogroups, global folds were largely conserved, whereas molecular divergence was primarily associated with localized structural variation in flexible loops and surface-exposed regions. Variability hotspots were significantly enriched near predicted cavities in several orthogroups, indicating that molecular diversification was concentrated in structurally permissive regions frequently associated with putative interaction surfaces. Stability analyses of reconstructed branch-specific substitutions revealed heterogeneous energetic effects, ranging from stabilizing to strongly destabilizing changes, while global protein architecture remained largely conserved. Together, these results suggest that evolutionary divergence in *Cereus* lineages is primarily associated with localized modifications of surface-accessible regions rather than large-scale structural innovation. More broadly, our findings demonstrate how integrating molecular evolutionary analyses with structural bioinformatics provides a robust framework for interpreting candidate adaptive genes in non-model organisms, while highlighting both the potential and limitations of structure-informed evolutionary inference.

**Supplementary Information:**

The online version contains supplementary material available at https://doi.org/10.1007/s10709-026-00293-4.

## Introduction

Understanding how organisms adapt to diverse and changing environments is a central goal of evolutionary biology (Jensen et al. 2007). At the molecular level, identifying how adaptive genetic changes contribute to molecular evolution and ultimately affect protein structure and function remains one of the major challenges in evolutionary genomics (Liberles et al. 2012; Edwards et al. 2022; Jayaraman et al. 2022). Adaptive substitutions can influence enzymatic activity, regulatory interactions, and responses to environmental stress (López-Maury et al. 2008; Vogt 2022), but statistical signatures of positive selection alone often provide limited insight into their structural and functional consequences. Advances in genomics and comparative phylogenetic methods have enabled the identification of genes evolving under positive selection (PS) across lineages, providing valuable insights into the genetic basis of adaptation (Amaral et al. 2024). However, translating these evolutionary signals into biologically meaningful interpretations remains a major challenge.

Branch-site models and related likelihood-based approaches based on the nonsynonymous/synonymous rate ratio (d_N_/d_S_; Yang 2007; Sun and Kozai 2024) have become widely used to detect positive selection acting on specific evolutionary lineages. These methods have been applied to diverse taxa and ecological contexts, revealing genes potentially involved in environmental adaptation, stress tolerance, and niche specialization (Amaral et al. 2024; Ma et al. 2025). However, although d_N_/d_S_ is a relevant indicator of selective pressure at the protein level, signatures of PS at the DNA level do not necessarily translate into functional divergence. Many amino acid substitutions may be selectively neutral or buffered by structural constraints, meaning that statistical signals alone provide limited information about their biological relevance (Ng and Henikoff 2006).

Recent advances in structural bioinformatics, particularly protein structure prediction using AlphaFold (Jumper et al. 2021), have created new opportunities for interpreting patterns of molecular evolution beyond sequence-based analyses alone (Varadi and Velankar 2023), helping to bridge the gap between statistical signatures of selection and their functional consequences. High-confidence structural models can now be generated for thousands of proteins, enabling comparative analyses that were previously restricted to a small number of experimentally solved structures. These developments allow researchers to integrate evolutionary, structural, and functional perspectives in a unified analytical framework (Varadi and Velankar 2023; Zhao et al. 2024).

Despite these advances, relatively few studies have integrated genome-wide molecular evolutionary analyses with comparative structural approaches across multiple lineages (e.g., Orsi et al. 2008; Batarseh et al. 2023; Ren et al. 2025). Most studies either identify candidate genes evolving under positive selection without investigating their structural implications or perform structural analyses without explicitly placing them within an evolutionary framework. Moreover, many existing studies rely on codon-aware alignments and residue-level mapping of positively selected sites, approaches that require complete coding sequences and therefore cannot be readily applied to transcriptome-based datasets, which frequently contain fragmented or partial transcripts. Consequently, integrating evolutionary signals with structural inference at a large scale remains technically challenging, particularly for non-model organisms (Schell et al. 2025).

*Cereus fernambucensis* Lem. and *C. insularis* Hemsl. (Cereeae, Cactaceae) compose a monophyletic group occurring in xeric coastal habitats within the Brazilian Atlantic Forest phytogeographic domain. *Cereus fernambucensis* is distributed in the restinga vegetation along the Brazilian Atlantic coast, whereas *C. insularis* is a narrow endemic restricted to coastal rocky outcrops of the Fernando de Noronha Archipelago. The phylogenetic relationships among populations of both species suggest that *Cereus insularis* originated from northernmost *C. fernambucensis* populations under a progenitor-derivative speciation model (Franco et al. 2024). In a previous comparative transcriptomic and phylogenomic study, Amaral et al. (2024) used branch-site models to identify multiple orthogroups showing signatures of lineage-specific positive selection in the *C. fernambucensis*-*C. insularis* clade. These findings suggested that positive selection may have contributed to shaping molecular variation across island and continental populations. However, the molecular and structural consequences of these evolutionary patterns remain largely unexplored. For example, even in the absence of codon-level site mapping, do genes exhibiting PS signatures show non-random patterns of structural divergence among lineages? In particular, it remains unclear whether these candidate positive-selection signals are associated with changes in overall protein fold, localized remodeling of surface-accessible regions potentially relevant to molecular interactions, or subtle modifications that preserve overall structural stability. To address this question, we used orthogroups previously identified as candidate genes evolving under positive selection (Amaral et al. 2024) to investigate how molecular evolutionary patterns are reflected in protein structural variation through comparative structural modeling, sequence variability analyses, pocket detection, and stability inference. Rather than directly inferring function, our objective was to evaluate whether structural analyses can provide additional evolutionary interpretation for candidate adaptive genes identified through molecular evolutionary analyses. Using structure predictions and quantitative structural comparison methods, we examine how evolutionary divergence was reflected in three-dimensional protein structures across multiple lineages. Specifically, we aim to: (i) assess the consistency of predicted structures among orthologous proteins; (ii) quantify structural divergence using objective metrics; (iii) identify regions of elevated sequence variability and evaluate their spatial distribution within protein structures; (iv) test whether variable regions form spatial clusters near predicted functional cavities; and (v) estimate the potential impact of lineage-specific substitutions on protein stability in high-confidence structural regions.

## Methods

### Dataset and selection of positively selected orthogroups

The dataset analyzed in this study was derived from a previously published transcriptomic analysis of adaptive evolution in the *C. fernambucensis-C. insularis* clade and related lineages (Amaral et al. 2024). That study identified candidate genes evolving under lineage-specific positive selection using branch-site models. Orthogroups exhibiting statistically significant likelihood ratio tests after false discovery rate correction and supported by Bayes Empirical analyses were considered candidate genes evolving under positive selection using PAML v.4.9 (Yang 2007). Five orthogroups previously identified as candidates for lineage-specific positive selection (OG0028003, OG0028976, OG0029756, OG0030099, and OG0031271) were selected for downstream predictive structural analyses. To explicitly connect the branch-site signals reported by Amaral et al. (2024) with protein-level molecular changes, we reconstructed amino acid states along the corresponding foreground branches and identified substitutions occurring specifically on the branches tested for positive selection. This distinction is important because branch-site evidence supports positive selection at the gene/branch level, rather than implying that every amino acid substitution occurring on that branch was itself positively selected. Therefore, branch-specific substitutions were treated as lineage-specific molecular changes associated with a branch showing evidence of positive selection, rather than as individually selected sites. These substitutions were subsequently mapped onto the predicted protein structures and evaluated according to local model confidence and, where appropriate, their predicted effects on protein stability. These orthogroups encode proteins associated with core cellular metabolism, stress response pathways, and regulatory processes as inferred from functional annotations reported in Amaral et al. (2024), which are frequently reported as targets of adaptive evolution in insular and environmentally heterogeneous systems (Wek et al. 2023).

### Retrieval and curation of orthologous protein sequences

Orthologous amino acid sequences corresponding to the selected orthogroups were retrieved from the output of OrthoFinder v.2.5.5 (Emms and Kelly 2019) and from curated protein predictions derived from transcriptomes. Protein sequences were obtained from datasets associated with each lineage. For each orthogroup, all available orthologs were extracted and manually inspected to remove truncated, low-complexity, or poorly aligned sequences. Redundant sequences were filtered using CD-HIT v.4.8.1 (Li and Godzik 2006) with an identity threshold of 100% to ensure non-redundant datasets. Sequence identifiers were standardized to reflect orthogroup membership and lineage origin. The final curated datasets were used as input for subsequent analyses.

The final datasets consisted of seven orthologous sequences per orthogroup. Mean pairwise amino acid identity ranged from 98.1 to 99.5% among orthologs (Table 1), confirming the high sequence conservation expected for single-copy orthogroups and providing a suitable framework for fine-scale structural comparisons.

**Table 1:** Evolutionary and structural characteristics of the analyzed single-copy orthogroups, including sequence representation, mean amino acid identity, structural confidence metrics, predicted disorder, and pairwise structural similarity among orthologous models

Orthogroup	Annotation	InterPro/GO	PSG branch(es)	Mean identity (%)	Mean pLDDT	Fraction pLDDT ≥70	Fraction disordered	Mean pairwise RMSD (Å)	Mean pairwise TM-score	Best experimental structural match (PDB)	Experimental structural support	Best AlphaFold DB match	AlphaFold DB structural context
OG0028003	S-adenosylmethionine decarboxylase proenzyme	–	B7	99.08	81.72	0.81	0.15	2.15	0.9	1MHM, plant S-adenosylmethionine decarboxylase	Strong, broad homologous support; experimentally characterized plant protein	AF-O24575-F1-model_v6, S-adenosylmethionine decarboxylase proenzyme	Strong, near-full-length match; 52.3% identity, E = 2.02 × 10⁻^53^
OG0028976	Organelle RRM domain-containing protein 2, mitochondrial-like	AdipoR/Haemolysin-III-related (IPR004254); RNA binding (GO:0003723)	B2, B3, B7	98.14	78.26	0.59	0.44	2.28	0.68	2RRB, Tra2β RNA-binding domain; additional RRM structures	Strong domain-level support; multiple experimentally determined RRM structures recovered	AF-Q9FIU6-F1-model_v6, organelle RRM domain-containing protein 2, mitochondrial	Strong, essentially full-length family match; 60.9% identity, E = 7.49 × 10⁻^1^⁹
OG0030099	F-box domain-containing protein	Protein binding (GO:0005515)	B4	98.65	69.24	0.59	0.92	2.21	0.93	7T1Y, Fbw7–Skp1–Myc degron complex	Moderate structural support; broad correspondence but low sequence identity	AF-Q9LMR4-F1-model_v6, putative F-box/kelch-repeat protein At1g15680	Broad family-level structural correspondence; 15.3% identity, E = 3.16 × 10⁻^1^⁶
OG0031271	Uncharacterized protein LOC110684135 [Chenopodium quinoa]	–	B7	99.47	61.00	0.34	1	4.42	0.31	No significant PDB100 match detected	No experimental structural support identified; low-confidence/disordered case	No match recovered	No corresponding AlphaFold/Swiss-Prot structural match detected
OG0029756	Heavy metal-associated isoprenylated plant protein	–	B4	98.39	48.76	0.2	0.81	5.35	0.35	2CRL, HMA/copper-binding domain	Domain-level support only; structural similarity restricted to the HMA region	AF-Q9LF57-F1-model_v6, heavy metal-associated isoprenylated plant protein 21	Domain-level match; 40.0% identity over ~22% of the query, E = 1.89 × 10⁻⁸

### Multiple sequence alignment, variability analysis, and mapping of branch-specific amino acid substitutions

Multiple sequence alignments were generated at the amino acid level using MAFFT v7.505 (Katoh et al. 2005) with the L-INS-i strategy, which is optimized for accuracy in datasets with low to moderate sequence divergence. Default gap opening and extension penalties were applied. Alignment quality was assessed visually and by summary statistics, including gap frequency and alignment length. To quantify site-specific variability, Shannon entropy (Shannon 1948) was calculated for each alignment position using custom Python scripts built with BioPython and SciPy. Positions with gap frequencies greater than 20% were excluded from entropy analyses. Sequence variability hotspots were defined as positions within the upper 5th percentile of entropy values among all non-gapped alignment positions.

For each orthogroup, aligned amino acid states were compared across the phylogenetic topology used in the selection analyses. Substitutions were assigned to specific foreground branches only when ancestral and derived states could be unambiguously reconstructed from the lineage distribution. Branch-specific substitutions were then mapped onto the corresponding predicted protein structures, and residue-level pLDDT scores were used to assess local structural confidence.

### Structural analyses of candidate adaptive genes

Three-dimensional protein structures were predicted using AlphaFold (Jumper et al. 2021; available at https://alphafoldserver.com/), an optimized high-throughput modeling tool. For each orthogroup and lineage, the corresponding amino acid sequence was submitted to AlphaFold using default parameters. For each protein, the model with the highest average predicted Local Distance Difference Test (pLDDT) score was selected for downstream analyses. Model confidence was evaluated using residue-level pLDDT scores. Regions with mean pLDDT values below 70 were considered low-confidence and were interpreted with caution. All protein structures analyzed in this study were predicted and evaluated as monomers. The structural comparisons, pocket analyses, residue mapping, and FoldX calculations therefore refer to monomeric models and should not be interpreted as predictions of biologically relevant oligomeric assemblies. The AlphaFold Protein Structure Database (AlphaFold DB; EMBL-EBI) was additionally queried using Foldseek against the AlphaFold/Swiss-Prot structural collection to determine whether previously predicted structures were available for proteins belonging to the same annotated families as the five selected orthogroups. These searches were used solely as external structural context for interpreting our results rather than as replacements for the lineage-specific models generated and analyzed here.

### Quantitative structural comparison and similarity assessment

Structural similarity among orthologous models was assessed using TM-align (Zhang and Skolnick 2005). Pairwise structural alignments were performed for all lineage pairs within each orthogroup. For each alignment, TM-score and root-mean-square deviation (RMSD) values were recorded. TM-score values were interpreted according to standard guidelines, with scores above 0.5 indicating shared global folds and values above 0.7 reflecting high structural similarity. RMSD values were calculated over aligned Cα atoms and used as a complementary measure of structural divergence. Results were compiled into pairwise similarity matrices and visualized as heatmaps using Python-based plotting libraries.

### Identification of structural cavities and pocket-hotspot association

Putative ligand-binding pockets were predicted using FPocket v4.0 (Le Guilloux et al. 2009) with default parameters. For each structural model, FPocket was used to identify cavities based on alpha sphere detection and Voronoi tessellation. Detected pockets were ranked by fpocket score, volume, and hydrophobicity. These predictions represent geometrically defined cavities and should not be interpreted as direct evidence of functional binding sites. To assess the spatial relationship between predicted pockets and sequence-variability hotspots, hotspot residues were mapped onto corresponding structural coordinates. Hotspot residues were classified as pocket-associated when at least one heavy atom was located within 5 Å of a pocket-defining alpha sphere. The spatial association between variability hotspots and pocket-associated residues was summarized using odds ratios comparing hotspot and non-hotspot occurrence in pocket-associated regions. Odds-ratio distributions across structural models were used as descriptive measures of hotspot-pocket association, with OR > 1 indicating greater relative hotspot representation near predicted cavities.

To provide an independent functional context for the predicted cavities, the functional annotations reported for each orthogroup were retrieved and compared with experimentally determined protein structures. Structural similarity searches against the Protein Data Bank (PDB) were performed using Foldseek v. 10-941cd33 (Van Kempen et al. 2024), and the highest-ranking structurally relevant hits were inspected for annotated catalytic, ligand-binding, or interaction-associated regions. When an experimentally characterized homolog was available, we evaluated the spatial relationship between lineage-specific substitutions, predicted pockets, and known structural or functional features. In cases where no sufficiently informative experimental homolog was identified, predicted cavities were retained strictly as geometrically defined structural features and were not assigned a specific functional role.

### In silico stability analysis

Protein stability was evaluated using FoldX v4 (Schymkowitz et al. 2005), which estimates changes in folding free energy (ΔΔG) associated with amino acid substitutions based on empirical force-field approximations and predicted structural models. Before the mutational analyses, all structural models were subjected to the RepairPDB function to optimize side-chain conformations and remove steric clashes. For each orthogroup, lineage-specific amino acid substitutions identified from multiple sequence alignments were introduced using the BuildModel command. For each mutation, five independent FoldX runs were performed, and mean ΔΔG values were calculated to minimize stochastic variation. Analyses were restricted to residues located in high-confidence structural regions (pLDDT > 70). Mutations in low-confidence regions were excluded. ΔΔG values were interpreted according to standard conventions, with positive values indicating destabilizing effects and negative values indicating stabilizing effects, while acknowledging the intrinsic uncertainty associated with force-field-based estimates and predicted backbones.

### Integration of structural, sequence, and functional analyses

Structural similarity metrics, entropy-based variability measures, pocket predictions, and stability estimates were integrated using custom Python scripts. For the purposes of this study, we operationally defined localized molecular divergence as the concentration of lineage-specific amino acid variation within restricted regions of otherwise highly conserved proteins, particularly when such variation occurred in structurally interpretable regions without extensive disruption of the conserved global fold. This pattern was evaluated by jointly considering sequence conservation, structural similarity, local structural confidence and disorder, the spatial distribution of variable and branch-specific sites relative to predicted cavities, and the predicted stability effects of reconstructed substitutions. Localized molecular divergence did not require every orthogroup to satisfy all of these features; rather, this integrative framework was used to distinguish well-supported cases of spatially restricted divergence from structurally ambiguous or methodologically limiting cases. The overall analytical workflow, which integrates evolutionary inference, structural modeling, functional site prediction, and stability analysis, is summarized in Figure S1.

## Results and discussion

### Selection of positively selected orthogroups and dataset structure

**Fig. 1:**
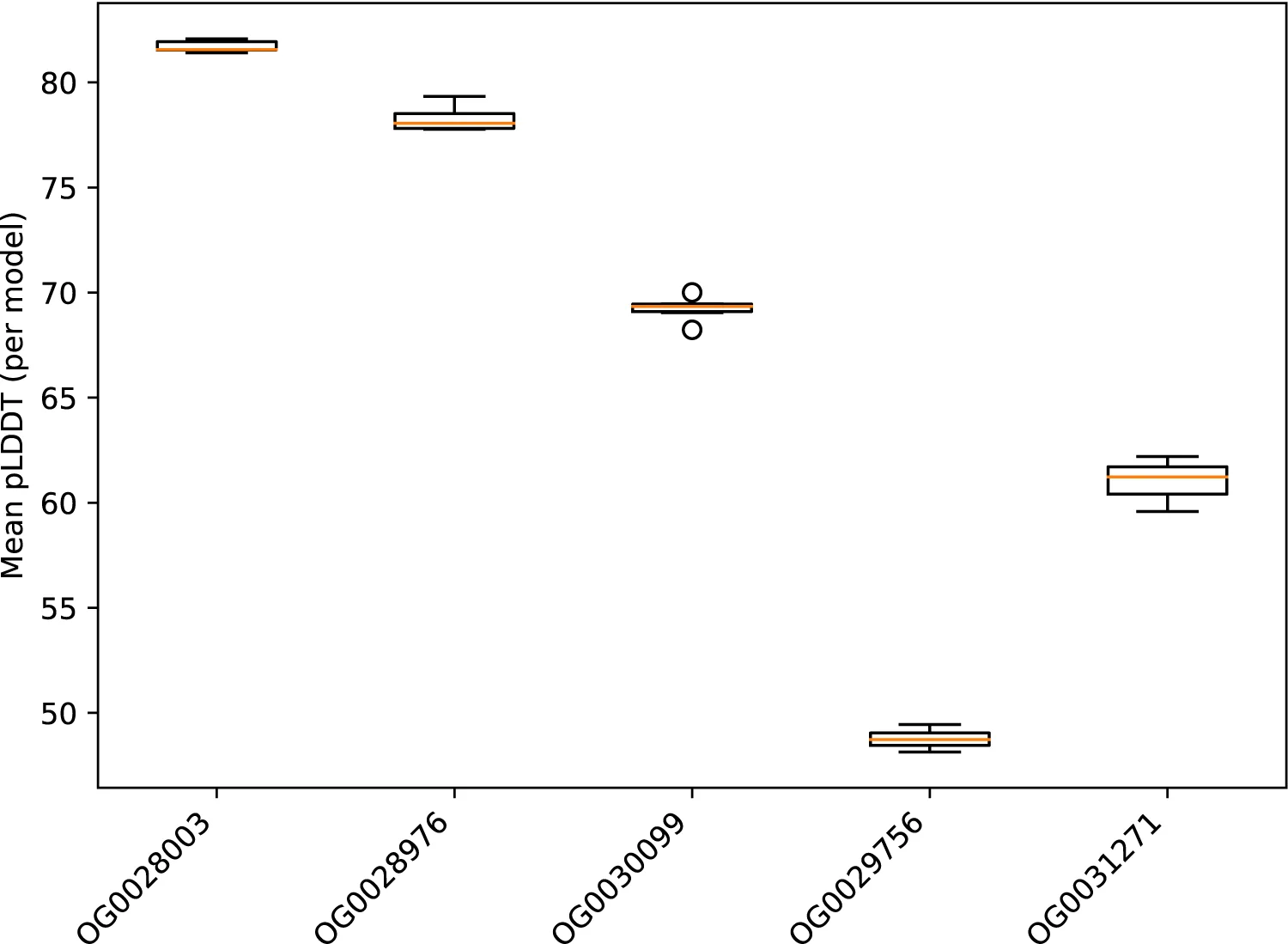
Distribution of residue-level AlphaFold confidence scores (pLDDT) across the analyzed orthogroups. Higher pLDDT values indicate greater confidence in the predicted local structural environment

The five selected orthogroups showed variable but generally interpretable structural quality across lineages, with two orthogroups displaying consistently high-confidence predictions and the remaining proteins exhibiting lower confidence or elevated predicted disorder (Fig. 1). These results support strong overall structural consistency among orthologous proteins, providing a reliable framework for interpreting lineage-specific molecular divergence (Objective i). Besides, indicate that the predicted models were structurally robust across lineages, providing a reliable basis for downstream comparisons of sequence variation, structural divergence, and stability. This approach is consistent with recent recommendations to incorporate AlphaFold confidence metrics into structure-informed evolutionary studies (Akdel et al. 2022).

The selected orthogroups represent candidate adaptive genes previously identified through molecular evolutionary analyses (Amaral et al. 2024) and include proteins functionally associated with metabolism and stress response, as defined by the annotation and selection framework reported by Amaral et al. (2024). This functional profile is compatible with evolutionary scenarios involving environmental heterogeneity, resource limitation, and fluctuating selective pressures. Island systems, in particular, often impose strong constraints on energetic efficiency and physiological resilience, thereby favoring the optimization of metabolic and regulatory networks (Whittaker and Fernández-Palacios 2007; Warren et al. 2015; Losos and Ricklefs 2009).

Moreover, comparative studies in vertebrates, insects, and plants have repeatedly shown that stress-related proteins and metabolic enzymes are recurrent targets of positive selection in insular and fragmented habitats (Jeffries et al. 2019; Tigano and Friesen 2016). Our results are consistent with this broader pattern and suggest that similar evolutionary processes may operate in our studied system. The selected orthogroups exhibited contrasting structural and evolutionary profiles (Table 1). Despite these differences, all orthogroups corresponded to single-copy genes represented across the seven analyzed lineages and displayed very high sequence conservation, with mean pairwise amino acid identities ranging from 98.1 to 99.5%. This high degree of sequence similarity indicates that the observed structural differences are associated with a relatively small number of lineage-specific substitutions rather than extensive sequence divergence.

Searches against the AlphaFold Protein Structure Database provided additional external structural context for four of the five orthogroups (Table 1). OG0028003 and OG0028976 showed strong, near-full-length matches to previously predicted proteins with annotations consistent with S-adenosylmethionine decarboxylase and RNA-recognition motif proteins, respectively. OG0029756 showed domain-level correspondence to heavy metal-associated isoprenylated plant proteins, whereas OG0030099 showed broad structural correspondence to predicted F-box/kelch-repeat proteins despite low sequence identity. No corresponding AlphaFold/Swiss-Prot structural match was recovered for OG0031271.

OG0028003 and OG0028976 showed the highest model confidence (mean pLDDT = 81.7 and 78.3, respectively), whereas OG0030099 also exhibited relatively high structural consistency despite slightly lower confidence values. These features make them the most suitable orthogroups for detailed structure-based functional interpretation. In contrast, OG0029756 and OG0031271 exhibited substantially lower model confidence (mean pLDDT = 48.8 and 61.0, respectively) and high predicted disorder (0.81 and 1.00, respectively), suggesting that these proteins may contain extensive intrinsically disordered regions. This pattern indicates that these latter orthogroups likely correspond to conformationally flexible or partially unstructured proteins. As a result, fine-scale structural inference is less reliable in these cases, highlighting an important methodological limit of structure-based analyses, thereby enabling subsequent assessments defined in Objectives (ii–v) (van Der Lee et al. 2014; Wright and Dyson 2015; Chen and Kriwacki 2018).

Accordingly, the high-confidence orthogroups were treated as the primary cases for structure-based evolutionary interpretation, whereas OG0029756 and OG0031271 were retained as methodological contrasts to evaluate the limits of structural inference in proteins containing extensive low-confidence or disordered regions.

### Structural model quality and lineage-specific structural divergence

Structural models were generated for all selected orthogroups, and residue-level profiles were used to assess local variation in model confidence (Jumper et al. 2021; Tunyasuvunakool et al. 2021). While overall structural robustness is described above (Objective i), here we focus on within-protein confidence patterns. OG0028003 and OG0030099 consistently displayed extensive high-confidence segments, with most residues exceeding pLDDT > 75 and relatively few low-confidence regions. These profiles are consistent with well-defined secondary and tertiary structural elements, enabling more reliable residue-level structural interpretation for these proteins (Akdel et al. 2022).

In contrast, the remaining orthogroups exhibited lower average pLDDT values and broader predicted intrinsically disordered regions (IDRs), thereby restricting the reliability of fine-scale structural and energetic analyses. Although lower-confidence regions should be interpreted cautiously, they may reflect biologically meaningful flexibility rather than prediction artifacts. Intrinsically disordered and flexible regions are common in regulatory proteins and stress-response factors and often associated with adaptive diversification (Wright and Dyson 2015; Babu 2016). Thus, localized low-confidence segments do not necessarily compromise the overall structural interpretation, but may instead indicate functional plasticity.

The distribution of pLDDT scores across orthogroups and lineages (Fig. 1) indicates that structural confidence was broadly consistent among populations. This pattern suggests that the structural differences inferred among lineages are unlikely to reflect systematic modeling bias. Integrating structural confidence metrics into evolutionary analyses has been increasingly advocated to reduce false functional inferences based solely on sequence-level signals (Akdel et al. 2022).

To quantitatively assess structural divergence among orthologous proteins, pairwise structural alignments were performed (Objective ii.). Across all orthogroups, pairwise comparisons revealed substantial variation in structural conservation among orthogroups. OG0028003 and OG0030099 exhibited consistently high TM-scores, whereas OG0031271 and OG0029756 showed markedly lower structural similarity. TM-scores above 0.5 typically reflect shared structural topology, whereas values above 0.7 indicate strong fold-level conservation (Zhang and Skolnick 2005). In this context, OG0028003 and OG0030099 showed consistently high TM-scores (> 0.7) and low RMSD values, revealing remarkable structural conservation despite underlying sequence divergence. Quantitatively, OG0028003 and OG0030099 exhibited low structural dispersion (mean RMSD = 2.15 Å and 2.21 Å, respectively), whereas OG0029756 and OG0031271 showed markedly higher divergence (mean RMSD = 5.35 Å and 4.42 Å), reflecting substantial conformational heterogeneity (Table 1). OG0028976 displayed intermediate behavior (mean TM-score = 0.683; RMSD = 2.28 Å), consistent with partial fold conservation combined with enhanced surface flexibility.

This pattern suggests strong functional constraints acting on core structural elements, consistent with the maintenance of essential functional architectures. Similar decoupling between sequence divergence and structural conservation has been widely reported and reflects the intrinsic robustness of protein folds (Chothia and Lesk 1986; Illergård et al. 2009; Liberles et al. 2012). The analyzed orthogroups exhibited extremely high amino acid identity (> 98% in all cases), indicating that the detected structural differences emerged despite very limited sequence divergence. This observation is consistent with the view that a small number of substitutions can contribute disproportionately to localized structural differentiation while preserving overall fold architecture. By contrast, orthogroups with lower TM-scores and higher RMSD values showed greater structural variability among lineages. Importantly, this divergence was primarily localized to loop regions and peripheral domains, whereas core secondary structure elements remained largely conserved (Bloom et al. 2006; Echave et al. 2016; Arenas et al. 2013).

Explicit mapping of amino acid changes onto the foreground branches provided a direct link between the previous branch-site analyses and the structural analyses performed here (Fig. 5; Table 3). Branch B4, corresponding to the ancestral lineage of the northern *C. fernambucensis* populations and *C. insularis*, contained lineage-specific substitutions in OG0029756 and OG0030099, whereas branch B7, corresponding to the *C. insularis* lineage, contained branch-specific substitutions in OG0028003 and OG0028976. These substitutions could therefore be positioned explicitly within the same phylogenetic context in which the positive-selection signals were originally detected. Importantly, we interpret these residues as substitutions occurring along branches showing evidence of positive selection, rather than as individually demonstrated positively selected sites (Venkat et al. 2018).

Structural confidence filtering further distinguished substitutions suitable for residue-level interpretation from those occurring in uncertain regions. In OG0028003, three branch-specific substitutions on B7 (D109N, A209G, and T254S) occurred in structurally reliable regions and could be evaluated using FoldX. In OG0029756, two B4 substitutions, L68S and S75P, were supported by high local structural confidence and were amenable to stability analysis. In OG0030099, the B4 substitutions R218S, A355P, and I377T were structurally mapped and evaluated, although confidence varied among sites and interpretations were therefore conditioned on local pLDDT. This branch-aware mapping eliminates the previous disconnect between gene-level evidence of positive selection and the protein structural analyses by explicitly identifying which molecular changes occurred along the corresponding evolutionary lineages.

**Fig. 2:**
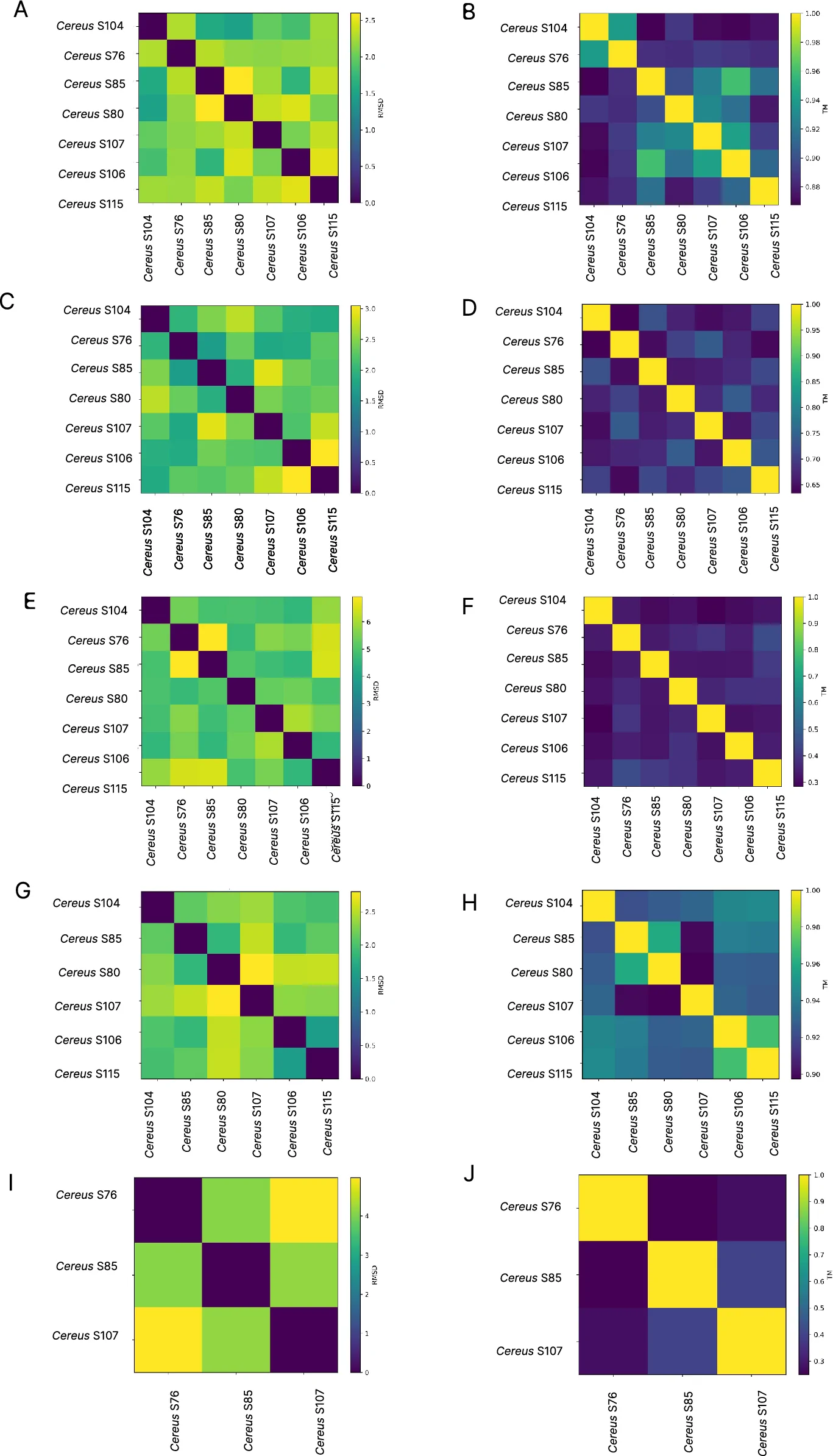
Heatmaps showing pairwise RMSD (Å) and TM-score values among predicted protein structures from selected *Cereus* orthogroups. **A**, **B**: OG0028003; **C**, **D**: OG0028976; **E**, **F**: OG0029756; **G**, **H**: OG0030099; **I**, **J**: OG0031271. RMSD indicates structural divergence, while TM-score reflects global fold similarity. Comparisons were performed among samples S104, S76, S85, S80, S107, S106, and S115

Heatmaps of pairwise RMSD and TM-score values (Fig. 2) further illustrate lineage-specific structural differentiation, with clearer divergence patterns observed in orthogroups previously identified as candidate genes evolving under positive selection. Together, these results show that integrating structural alignment metrics with evolutionary modeling provides a more nuanced view of evolutionary divergence, bridging genotype–structure–phenotype relationships (Liberles et al. 2012). Overall, the structural analyses indicate that evolutionary divergence in the studied system predominantly occurs through localized conformational adjustments rather than through large-scale fold innovation, reinforcing the view that protein evolution is typically constrained by fold stability while permitting functional modulation at peripheral regions (Ferreiro et al. 2025).

### Sequence variability hotspots and their functional-structural context

Shannon entropy analyses were implemented to identify regions of elevated sequence variability and evaluate their distribution along protein sequences (Objective iii.), without assuming direct evidence of positive selection at individual sites. Entropy values were calculated for each alignment position, and highly variable regions were defined as positions with top-5% entropy values and exhibiting low gap frequencies. Importantly, entropy-based hotspots reflect patterns of molecular sequence divergence and are not equivalent to codon-based signatures of positive selection. Across orthogroups, sequence variability hotspots were unevenly distributed along protein sequences and tended to cluster in flexible loops, terminal regions, and surface-exposed segments. In contrast, structurally conserved cores displayed consistently low entropy values, reflecting strong purifying constraints associated with fold stability and functional integrity (Chothia and Lesk 1986; Echave et al. 2016; Liberles et al. 2012).

This spatial segregation between conserved cores and more variable peripheral regions is a well-established hallmark of protein evolution. Core residues are typically constrained by thermodynamic and folding requirements, whereas surface-exposed regions tolerate higher mutational loads and may facilitate lineage-specific functional modulation, particularly when combined with independent evidence of positive selection (Bloom et al. 2006; Goldstein 2011; Nichol et al. 2019). Orthogroups with higher structural conservation exhibited fewer, more localized hotspots, whereas structurally divergent proteins displayed broader regions of elevated variability. This relationship indicates a tight coupling between fold stability and evolutionary flexibility, consistent with theoretical and empirical models of structure–sequence coevolution (Illergård et al. 2009; Arenas et al. 2013).

**Fig. 3:**
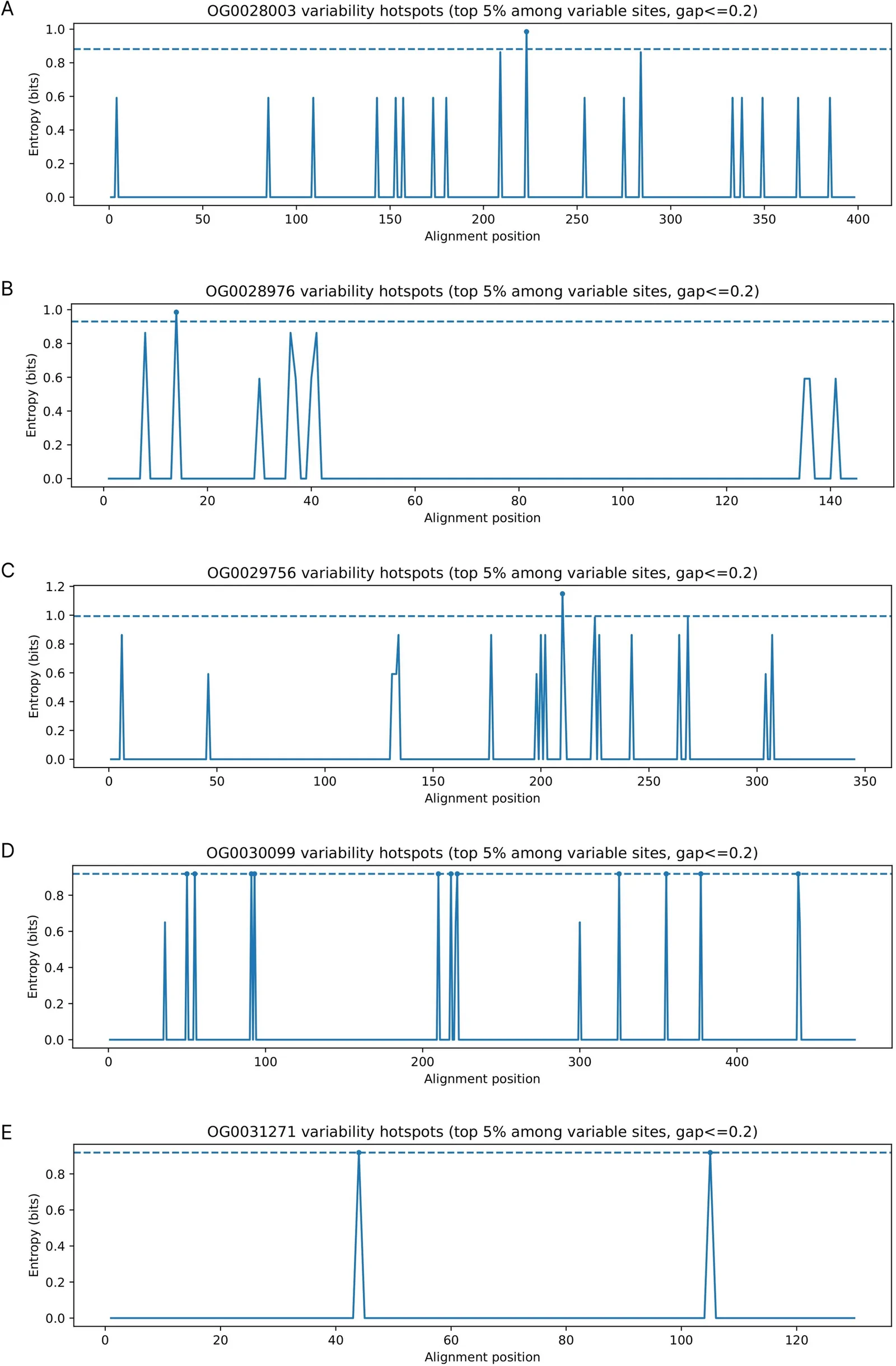
Shannon entropy profiles across multiple sequence alignments for selected orthogroups: **A** OG0028003, **B** OG0028976, **C** OG0029756, **D** OG0030099, and **E** OG0031271. Dashed lines indicate the top 5% most variable sites after excluding gap-rich positions. Peaks represent entropy-defined sequence variability hotspots

**Fig. 4:**
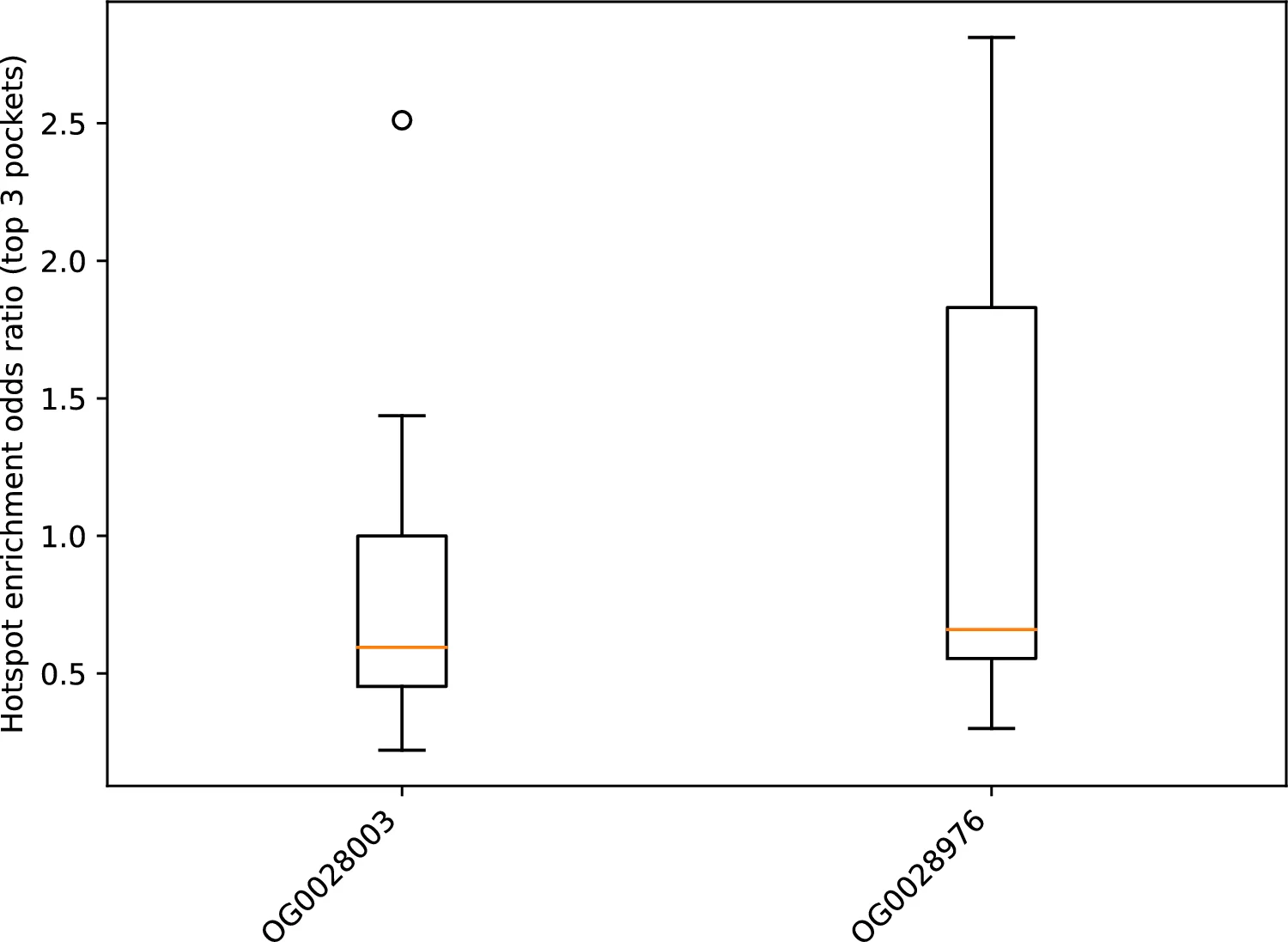
Boxplot showing hotspot enrichment odds ratios for entropy-defined sequence variability hotspots located within the top three predicted pockets identified by fpocket. Values greater than one indicate a higher-than-expected concentration of variable residues near predicted cavities

The entropy profiles and hotspot distributions (Figs. 3 and 4) revealed that molecular diversification is concentrated in structurally permissive regions rather than within the conserved structural core. This pattern is consistent with the general expectation that purifying selection preserves residues essential for structural stability, whereas diversification accumulates in flexible or solvent-exposed regions.

Several substitutions also involve shifts in physicochemical properties, including polarity, charge, and hydrophobicity, between continental and island lineages, potentially altering interaction surfaces or local binding environments. Together, these results indicate that sequence variability is spatially concentrated in structurally permissive regions rather than uniformly distributed across the protein architecture.

To evaluate the functional relevance of sequence variability, structural cavities, and their spatial relationships with entropy-defined hotspots were systematically assessed (Objective iv). For most orthogroups, major pockets were consistently detected across lineages, indicating that overall binding-site architecture is evolutionarily conserved at the geometric level. Such conservation is expected for functionally essential interaction surfaces and has been observed across diverse protein families (Cukuroglu et al. 2014; Konc and Janežič 2014; Gao et al. 2022). These conserved pockets were frequently located near regions compatible with ligand-binding or regulatory interfaces, suggesting potential functional relevance that remains to be experimentally validated. Descriptive analysis of hotspot–pocket spatial association revealed a tendency for entropy-defined variability hotspots to occur preferentially near predicted cavities in the two orthogroups for which enrichment estimates were available (OG0028003 and OG0028976; Fig. 4; Table 2). Odds-ratio distributions were generally centered at or above the null expectation of OR = 1, although substantial variation was observed among structural models. These results therefore suggest a non-uniform spatial relationship between sequence variability and predicted cavities, but do not provide evidence that variability hotspots are systematically or significantly enriched at functional sites. Rather, they indicate that localized sequence divergence can coincide with geometrically defined pocket-associated regions in at least some structurally interpretable orthogroups. This pattern is consistent with, but does not by itself demonstrate, functional or adaptive divergence. Similar hotspot-pocket associations have been reported in studies of enzyme specificity, protein–protein interactions, and signaling proteins, where amino acid substitutions can fine-tune binding properties without compromising fold stability (Aftab et al. 2024).

**Table 2:** Spatial association between entropy-defined sequence variability hotspots and predicted structural cavities

Orthogroup	Models analyzed	Pocket comparisons	Mean OR	Median OR	OR range	OR >1, n (%)	Minimum Fisher P	Minimum BH-FDR	Interpretation
OG0028003	7	21	0.804	0.595	0.221–2.511	5 (23.8%)	0.155	1,000	Heterogeneous association; no significant enrichment
OG0028976	7	21	1.114	0.660	0.300–2.812	7 (33.3%)	0.259	1,000	Heterogeneous association; no significant enrichment

Odds ratios and Fisher’s exact tests were calculated for the three highest-ranked fpocket cavities across seven structural models per orthogroup, with Benjamini–Hochberg correction for multiple testing. No significant enrichment was detected after correction; odds ratios are therefore interpreted descriptively

Comparison with experimentally determined PDB structures provided independent structural context for several orthogroups. OG0028003, annotated as an S-adenosylmethionine decarboxylase proenzyme, showed strong and recurrent similarity to experimentally determined AdoMetDC structures, including the experimentally characterized potato homolog (PDB 1MHM). OG0028976 showed consistent domain-level similarity to multiple experimentally resolved RNA-recognition motif proteins, supporting its RNA-binding annotation. OG0029756 matched several experimentally characterized heavy-metal-associated (HMA) domains, although these similarities covered only a limited portion of the full-length protein and were therefore interpreted as domain-level rather than global structural support. OG0030099 showed broad structural correspondence to Fbw7/F-box-related complexes, consistent with its F-box annotation, but this similarity occurred despite low sequence identity and was interpreted cautiously. In contrast, no significant PDB100 match was recovered for OG0031271, consistent with its low structural confidence and uncharacterized annotation. Together, these comparisons provide independent structural context for selected predicted cavities while reinforcing that cavity geometry alone does not establish functional equivalence. Mapping the branch-specific substitutions onto the 1MHM reference structure showed that T254S corresponded to a region close to the experimentally characterized catalytic environment, lying approximately 2.8 Å from His249 and 5.4 Å from the pyruvoyl cofactor. This spatial proximity supports the structural relevance of the region containing T254S, while not demonstrating a direct functional effect of the substitution.

From an evolutionary perspective, this spatial association is compatible with models in which lineage-specific sequence variation can accumulate near regions involved in intermolecular recognition, substrate affinity, or regulatory modulation (Peng et al. 2022). Such regions are expected to experience fluctuating selective pressures in heterogeneous environments and may therefore represent important substrates for evolutionary diversification (Arenas et al. 2013; Ding et al. 2022; Jimenez-Rosales and Flores-Merino 2018). Pocket proximity, solvent exposure, and conformational flexibility are often correlated structural properties. Consequently, the observed enrichment of variability hotspots near predicted cavities should not be interpreted as direct evidence that cavity-associated residues are the primary targets of evolutionary divergence. Instead, these results indicate that molecular diversification is preferentially concentrated in structurally permissive regions that frequently coincide with surface-accessible and pocket-associated areas. Distinguishing the relative contribution of these factors will require additional analyses and experimental validation.

In contrast, proteins displaying lower structural confidence showed weaker or inconsistent pocket-hotspot associations. This limitation likely reflects uncertainty in structural reconstruction rather than the genuine biological absence of functional coupling. These results emphasize the importance of integrating confidence metrics when interpreting structure–function relationships derived from predicted models (Akdel et al. 2022). The combined analysis of entropy profiles and pocket proximity (Fig. 4; Table 2) indicates that sequence variability can coincide with pocket-associated and surface-accessible regions in structurally interpretable orthogroups. This spatial pattern is consistent with the operational definition of localized molecular divergence used here, while avoiding the assumption that predicted cavities necessarily represent functional interaction sites. This convergence between sequence variability, evolutionary and structural context, and predicted cavity architecture supports the structural coherence of the evolutionary patterns identified in these candidate positive-selection genes.

### Structural stability and integrated patterns of evolutionary divergence

Across the four orthogroups containing reconstructed branch-specific substitutions, 22 amino acid changes were identified. Eight substitutions could be evaluated by FoldX, whereas five were excluded because they occurred in low-confidence structural regions and nine because no suitable ancestral-state structural model was available (Table 3; Table S1). Thus, stability calculations were restricted to substitutions for which the structural context was considered sufficiently interpretable, rather than to a subjectively selected set of representative mutations.

**Table 3:** Integrated evolutionary and structural characterization of lineage-specific substitutions

Orthogroup	Branch	Position/substitution	Evidence class	Consensus frequency (%)	Entropy (bits)	Local pLDDT	Mean ΔΔG (kcal·mol⁻^1^)	SD	Interpretation
OG0028003	–	Y85	Sequence-variable site	57.1	0.98	–	1.269	0.06	Mildly destabilizing
OG0028003	–	I223	Sequence-variable site	57.1	0.98	–	3.284	0.01	Strongly destabilizing hotspot
OG0028003	B7	D109N	Branch-specific substitution	–	–	93.18	−0.724	15	Stabilizing
OG0028003	B7	A209G	Branch-specific substitution	–	–	85.81	1	0	Destabilizing
OG0028003	B7	T254S	Branch-specific substitution	–	–	97.43	1.155	5	Destabilizing
OG0028976	–	G14	Sequence-variable site	57.1	0.98	–	1.305	0.06	Mild to moderate destabilization
OG0028976	–	H136	Sequence-variable site	51.7	0.92	–	−0.051	0.07	Near-neutral
OG0029756	–	D210	Sequence-variable site	71.4	1.1488	–	−0.140	0.01	Mildly stabilizing/variable
OG0029756	B4	L68S	Branch-specific substitution	–	–	97.53	5.0	0	Strongly destabilizing
OG0029756	B4	S75P	Branch-specific substitution	–	–	95.57	1.40	0	Destabilizing
OG0030099	–	C36	Sequence-variable site	66.7	0.92	–	−0.751	0.06	Mildly stabilizing
OG0030099	–	F50	Sequence-variable site	66.7	0.92	–	0.49	0.07	Weakly destabilizing
OG0030099	–	V55	Sequence-variable site	66.7	0.92	–	4.47	0.16	Strongly destabilizing
OG0030099	B4	R218S	Branch-specific substitution	–	–	67.93	1	0	Destabilizing; lower-confidence structural region
OG0030099	B4	A355P	Branch-specific substitution	–	–	75.05	−0.890	0	Stabilizing
OG0030099	B4	I377T	Branch-specific substitution	–	–	78.67	1.49	0	Destabilizing
OG0031271	–	E44	Sequence-variable site	66.7	0.92	–	0.3	0.01	Methodological control
OG0031271	–	S105	Sequence-variable site	66.7	0.92	–	−0.401	0.01	Methodological control

Shannon entropy (bits) and consensus frequencies were calculated from multiple sequence alignments, while stability effects (ΔΔG) were estimated using FoldX across independent replicates. Positive ΔΔG values indicate destabilization

**Fig. 5:**
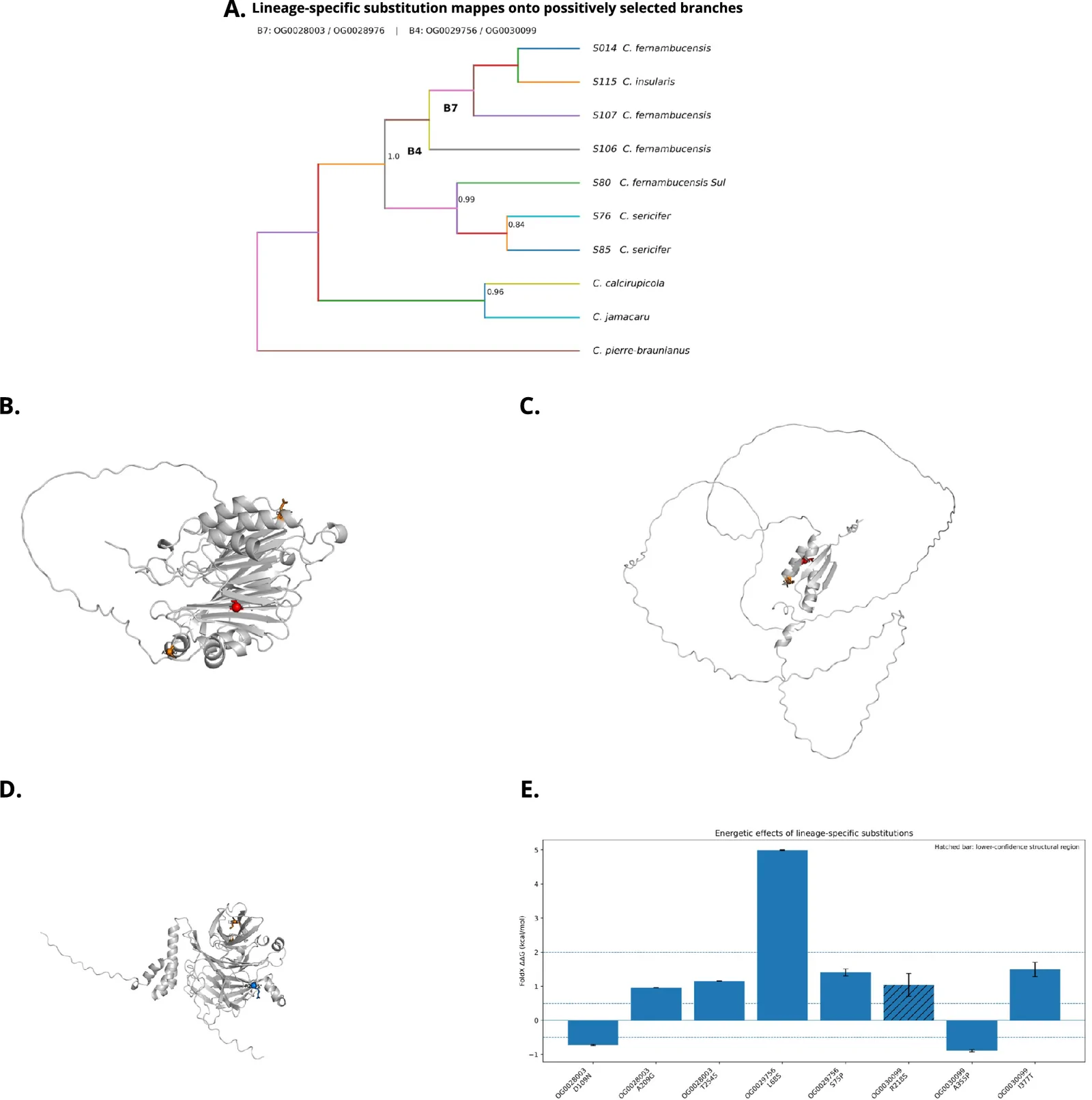
Integration of branch-specific evolutionary and structural evidence in selected orthogroups. **A** Phylogenetic context showing the foreground branches associated with lineage-specific positive-selection signals and the amino acid substitutions reconstructed along these branches. **B**–**D** Predicted protein structures of representative orthogroups with branch-specific substitutions mapped onto their structural coordinates. Sites selected for detailed energetic interpretation are highlighted according to local structural confidence. **E** FoldX ΔΔG estimates for branch-specific substitutions located in structurally interpretable regions. Positive values indicate predicted destabilization and negative values indicate predicted stabilization. The figure explicitly connects branch-level evolutionary signals with the amino acid changes occurring along the corresponding evolutionary lineages; individual substitutions are not interpreted as independent evidence of positive selection

Representative structural models were subjected to systematic mutational analysis of the reconstructed branch-specific substitutions, and changes in folding free energy were estimated using FoldX, providing comparative rather than absolute estimates of energetic effects (Objective v.). Structural confidence was explicitly incorporated into the interpretation because energetic estimates derived from uncertain predicted backbones should be treated cautiously (Jumper et al. 2021; Akdel et al. 2022). Among the eight branch-specific substitutions evaluated by FoldX, predicted stability effects were heterogeneous (Fig. 5; Table 3). The strongest destabilizing effect was observed for L68S in OG0029756 on branch B4 (mean ΔΔG = +4.991 ± 0.009 kcal·mol⁻^1^). Additional destabilizing effects were observed for S75P in OG0029756 (+ 1.404 ± 0.104 kcal·mol⁻^1^), T254S (+ 1.155 ± 0.005 kcal·mol⁻^1^) and A209G (+ 0.963 kcal·mol⁻^1^) in OG0028003, and I377T (+ 1.493 ± 0.213 kcal·mol⁻^1^) in OG0030099. Conversely, D109N in OG0028003 (− 0.724 ± 0.015 kcal·mol⁻^1^) and A355P in OG0030099 (− 0.890 ± 0.033 kcal·mol⁻^1^) were predicted to have stabilizing effects. R218S in OG0030099 showed a destabilizing estimate (+ 1.041 ± 0.333 kcal·mol⁻^1^), but its local structural confidence was below the predefined pLDDT >70 threshold (pLDDT = 67.93); therefore, this result was retained only as a lower-confidence exploratory estimate and was not given the same interpretive weight as substitutions located in high-confidence regions.

The branch-specific stability analysis did not reveal a uniform energetic direction of evolutionary change. Of the eight substitutions evaluated, five were predicted to be destabilizing, including one strongly destabilizing substitution (OG0029756 L68S), whereas two were stabilizing and one represented a lower-confidence destabilizing estimate. The coexistence of stabilizing, moderately destabilizing, and strongly destabilizing effects is compatible with the broader view that protein evolution occurs under structural and thermodynamic constraints, with mutational effects being accommodated provided that essential fold stability is maintained (Bloom et al. 2006; Goldstein 2011; Tokuriki and Tawfik 2009). More generally, the preservation of global protein architecture despite localized energetic perturbations is consistent with models of structurally constrained protein evolution in which evolutionary change can accumulate in structurally permissive regions without requiring extensive remodeling of the conserved protein scaffold (Liberles et al. 2012; Arenas et al. 2013; Echave et al. 2016). Importantly, the substitutions analyzed here were reconstructed on the same foreground branches associated with the gene-level positive-selection signals, thereby directly connecting the evolutionary and structural analyses. Nevertheless, because branch-site evidence applies to the gene and foreground branch rather than demonstrating selection on each individual residue, the energetic effects reported here should be interpreted as structural consequences of lineage-specific substitutions occurring along branches with evidence of positive selection, rather than as evidence that the individual substitutions were themselves positively selected.

When examined individually, the analyzed orthogroups revealed complementary evolutionary trajectories. OG0028003 represented a highly conserved structural scaffold with localized surface diversification and generally moderate predicted energetic effects, consistent with localized divergence without major architectural disruption. OG0030099 exhibited similar global fold conservation but greater localized surface variability, consistent with spatially restricted evolutionary divergence in structurally accessible regions. OG0028976 displayed intermediate structural conservation combined with elevated disorder, indicating enhanced regulatory or signaling flexibility. In contrast, OG0029756 showed low structural confidence and extensive disorder, restricting detailed functional inference, whereas OG0031271 primarily served as a methodological reference, illustrating the limits of structure-based evolutionary interpretation in highly flexible and structurally uncertain systems.

Beyond the specific biological patterns observed in the studied orthogroups, our analyses illustrate a framework for integrating molecular evolutionary inference with structural bioinformatics to improve the interpretation of candidate adaptive genes. In this approach, statistical evidence of lineage-specific positive selection identifies candidate genes, while subsequent analyses place amino acid substitutions within their structural and biophysical context. Specifically, sequence variability is mapped onto predicted protein structures to evaluate whether divergence concentrates in structurally permissive regions or near interaction-associated surfaces, and stability analyses assess whether substitutions occur within thermodynamically tolerable regimes. This framework links positive selection signals, amino acid substitutions, structural context, and potential structural and functional implications, thereby bridging sequence-level evolutionary inference with protein structure and biophysical constraints (Liberles et al. 2012; Echave et al. 2016; Jayaraman et al. 2022).

By integrating structural similarity metrics (Objective ii), sequence variability profiles (Objective iii), pocket enrichment analyses (Objective iv), and stability estimates (Objective v), within the framework of structurally consistent models (Objective i), we identified consistent patterns linking evolutionary divergence to protein architecture. Orthogroups previously identified as candidate targets of lineage-specific positive selection tended to display localized structural divergence near surface-accessible and potentially interaction-associated regions. These regions were characterized by elevated sequence entropy, enrichment near predicted interaction pockets, and moderate effects on stability, with occasional pronounced destabilization at specific hotspots, collectively suggesting localized remodeling of molecular interaction surfaces. Such coordinated patterns are consistent with contemporary models of protein evolution in which evolutionary diversification is frequently concentrated at functional interfaces rather than distributed uniformly across structures (Nichol et al. 2019; Pillai et al. 2022). This mode of evolution enables fine-tuning of binding specificity, regulatory responsiveness, or interaction networks while maintaining global fold stability. In contrast, highly conserved orthogroups exhibited low sequence variability, strong structural conservation, and minimal stability perturbations, indicating stronger functional and biophysical constraints. These proteins likely occupy central positions in essential cellular pathways, where even minor perturbations may incur substantial fitness costs (Liberles et al. 2012; Echave et al. 2016).

The observed coupling between evolutionary signals, structural context, and energetic effects supports the biological relevance of the detected patterns. The results are consistent with non-random evolutionary differentiation of protein surfaces under lineage-specific ecological and physiological contexts. The integrative analyses further suggest that evolutionary divergence in the analyzed lineages is predominantly associated with localized remodeling of protein surfaces, mediated by coordinated patterns of sequence variability, structural configuration, and thermodynamic stability. Together, these observations support the view that protein evolution can proceed through incremental modifications of structurally permissive regions while preserving overall fold architecture and global stability. (Arenas et al. 2013; Liberles et al. 2012; Jayaraman et al. 2022).

The multiple lines of evidence obtained in this study converge toward a consistent evolutionary scenario despite the inherent limitations associated with large-scale structure-informed analyses. The structural models generated by AlphaFold showed high confidence for the principal proteins discussed here, allowing comparative analyses of structural conservation, sequence variability, pocket distribution, and energetic effects. Although predicted cavities represent geometrically defined structural features and require experimental validation to confirm specific molecular functions, their recurrent association with regions of elevated sequence divergence provides an additional layer of biological context for interpreting evolutionary patterns. Likewise, while the present analyses do not directly test the functional consequences of individual substitutions, the concordance among evolutionary, structural, and biophysical metrics supports the interpretation that lineage-specific divergence is preferentially concentrated in structurally permissive and interaction-associated regions of these proteins.

### Surface-centered evolutionary divergence across lineages

When considered jointly, the structural, evolutionary, and biophysical analyses reveal a consistent pattern linking lineage-specific divergence to protein spatial organization. Across orthogroups, variability hotspots and lineage-specific substitutions are predominantly concentrated in surface-exposed regions and flexible loops rather than within structurally constrained cores (Figs. 3 and 4). The majority of high-entropy sites occur in loop regions or solvent-exposed surfaces, reinforcing the observation that diversification is largely restricted to structurally permissive regions. This spatial segregation between conserved cores and variable peripheral regions is consistent with established models of protein evolution in which purifying selection maintains residues essential for fold stability whereas diversification accumulates in solvent-accessible segments that can tolerate higher mutational loads (Chothia and Lesk 1986; Bloom et al. 2006; Echave et al. 2016; Liberles et al. 2012).

Variability hotspots showed a tendency to occur near predicted structural cavities in the orthogroups for which hotspot–pocket association could be evaluated (Fig. 4; Table 2). Odds-ratio distributions varied among models, indicating that this spatial correspondence was heterogeneous rather than universal. This pattern supports the view that localized sequence divergence can overlap with structurally permissive, pocket-associated regions without implying that these cavities constitute experimentally validated functional sites. Such coupling between sequence variability and predicted interaction surfaces suggests that evolutionary divergence is concentrated near molecular interfaces rather than structurally critical cores. Stability analyses further support this interpretation, as most lineage-specific substitutions produced mild or near-neutral energetic effects in high-confidence structural regions (Table 3), consistent with evolutionary change occurring within thermodynamically permissive regimes (Tokuriki and Tawfik 2009; Bloom et al. 2006; Goldstein 2011).

These results indicate that evolutionary diversification is concentrated in surface-exposed regions that frequently overlap with putative interaction-associated surfaces identified by geometric cavity detection, rather than in structurally critical cores. This pattern is consistent with localized evolutionary differentiation occurring in structurally permissive regions and, although compatible with adaptive functional divergence, does not by itself demonstrate adaptive change (Liberles et al. 2012; Echave et al. 2016; Jayaraman et al. 2022).

The integrated comparison revealed that localized molecular divergence was most strongly supported for OG0028003 and OG0030099. Both proteins combine very high sequence conservation (99.08% and 98.65%, respectively) with strong conservation of the predicted global fold (mean pairwise TM-scores of 0.901 and 0.933), while reconstructed lineage-specific substitutions are restricted to a comparatively small number of positions. In contrast, OG0029756 and OG0031271 showed extensive predicted disorder and substantially lower structural conservation, limiting residue-level structural interpretation and supporting their treatment as methodological contrasts rather than equivalent evidence for localized structural divergence. OG0028976 occupied an intermediate position, with high sequence conservation and experimental domain-level support but greater predicted disorder and lower structural conservation. Thus, the five orthogroups do not represent equally strong examples of localized molecular divergence; rather, they span a continuum of structural interpretability that explicitly defines both the applicability and the limits of the proposed framework (Table 4).

**Table 4:** Integrative evidence for localized molecular divergence across the five orthogroups

Orthogroup	Identity (%)	Mean TM-score	Mean pLDDT	Disorder fraction	PSG branch(es)	Branch-specific substitutions	FoldX assessment	PDB structural context	Integrated interpretation
OG0028003	99.08	0.901	81.72	0.147	B7	4	3/4 evaluable; 1 stabilizing, 2 destabilizing	Strong; experimental AdoMetDC homologs, including plant structure	Strongly supported localized divergence
OG0028976	98.14	0.683	78.26	0.439	B2, B3, B7	1	Not evaluated; substitution in low-confidence region	Strong domain-level RRM support	Localized divergence with structural-confidence limitation
OG0030099	98.65	0.933	69.24	0.92	B4	6	3/6 evaluable; 1 stabilizing, 2 destabilizing	Moderate; Fbw7/F-box-related structural correspondence	Strongly supported localized divergence, with site-specific confidence limitations
OG0029756	98.39	0.346	48.76	0.810	B4	11	2/11 evaluable; 1 destabilizing, 1 strongly destabilizing	HMA domain-level support only	Methodological contrast: extensive structural uncertainty
OG0031271	99.47	0.308	61.00	1.000	B7	None reconstructed	Not suitable for robust FoldX assessment	No significant PDB100 match	Methodological limit / structurally unresolved case

## Conclusions

By integrating evidence of positive selection with structural modeling, sequence-variability analyses, pocket detection, and stability inference, this study provides an integrative framework for interpreting molecular evolutionary divergence within the *C. fernambucensis-C. insularis* clade. Our results show that lineage-specific molecular divergence is predominantly associated with localized remodeling of surface-exposed and interaction-associated regions, particularly in orthogroups exhibiting high structural confidence, while highly flexible proteins highlight the current limits of integrating structural information into molecular evolutionary inference. This pattern suggests that functional diversification may be facilitated by subtle modifications to molecular interfaces rather than by large-scale structural rearrangements. Methodologically, our integrative approach demonstrates that combining AlphaFold confidence metrics with structural, energetic, and evolutionary analyses improves the evolutionary interpretation of candidate genes identified through positive-selection analyses. Although future studies incorporating experimental structural and functional validation will be important to refine these interpretations, the congruent patterns recovered across independent analytical approaches indicate that integrating molecular evolutionary analyses with structural bioinformatics provides valuable insights into the molecular basis of lineage divergence in non-model organisms. By integrating molecular evolutionary analyses with structural bioinformatics, this framework provides a robust and broadly applicable strategy for interpreting candidate adaptive genes and investigating lineage-specific molecular divergence across non-model organisms.

## Supplementary Information

Below is the link to the electronic supplementary material.

Supplementary file1 (DOCX 6495 KB)

Supplementary file2 (XLSX 7 KB)

## Data Availability

Custom scripts and detailed file descriptions are available in GitHub (https://github.com/BBMDO/CeEVoS).
