# Correction: Integrating evolutionary signals and predicted protein structure reveals localized molecular divergence in *Cereus* lineages

**DOI:** 10.1007/s10709-026-00301-7

**Published:** 2026-11-13

**Authors:** Danilo T. Amaral, João Alfredo Teodoro, Maria Izadora Oliveira Cardoso, Evandro Marsola Moraes, Fernando Faria Franco, Isabel A. S. Bonatelli

**Affiliations:** 1 Centro de Ciências Naturais e Humanas, Universidade Federal do ABC (UFABC), Avenida dos Estados, 5001, Bloco A, 504-3 Room, Santo André,, SP 09210-580 Brazil; 2 Departamento de Biologia, Centro de Ciências Humanas e Biológicas, Universidade Federal de São Carlos (UFSCar), Sorocaba, Brazil; 3 Químicas e Farmacêuticas, Departamento de Ecologia e Biologia Evolutiva, Universidade Federal de São Paulo, Instituto de Ciências Ambientais, Diadema, SP, Brazil; 4 Químicas e Farmacêuticas, Programa de Pós-Graduação em Ecologia e Evolução, Universidade Federal de São Paulo, Instituto de Ciências Ambientais, Diadema, SP, Brazil

**Correction: Genetica (2026) 154:42**

https://doi.org/10.1007/s10709-026-00293-4

In this article the author’s name João Alfredo Teodoro was incorrectly written as João Alfredo Teodo.

The original article has been corrected.

