# Supplementary material for: Integrating evolutionary signals and predicted protein structure reveals localized molecular divergence in Cereus lineages
Source: Genetica. 2026 Sep 25;154(1):42. doi: 10.1007/s10709-026-00293-4 (PMC13615019; doi:10.1007/s10709-026-00293-4)
Supplement: Supplementary file 1 [file 10709_2026_293_MOESM1_ESM.docx]

**Integrating evolutionary signals and predicted protein structure reveals localized molecular divergence in *Cereus* lineages**

Danilo T. Amaral^1,*^, João Alfredo Teodo^1^, Maria Izadora Oliveira Cardoso^1^, Evandro Marsola Moraies^2^_,_ Fernando Faria Franco^2^, and Isabel A.S. Bonatelli^3,4^

^1^Centro de Ciências Naturais e Humanas, Universidade Federal do ABC (UFABC), Santo André, São Paulo, Brazil.

^2^Departamento de Biologia. Centro de Ciências Humanas e Biológicas. Universidade Federal de São Carlos (UFSCar). Sorocaba, Brazil.

^3^Universidade Federal de São Paulo. Instituto de Ciências Ambientais, Químicas e Farmacêuticas. Departamento de Ecologia e Biologia Evolutiva, Diadema, São Paulo, Brazil.

^4^Universidade Federal de São Paulo. Instituto de Ciências Ambientais, Químicas e Farmacêuticas. Programa de Pós-Graduação em Ecologia e Evolução, Diadema, São Paulo, Brazil.

^*^Corresponding author, Avenida dos Estados, 5001, bloco A, 504-3 room. ZIP CODE 09210-580, Santo André, SP, Brazil. email: [danilo.trabuco@ufabc.edu.br](mailto:danilo.trabuco@ufabc.edu.br)

**Figure S1.** Integrative pipeline for detecting and interpreting adaptive protein evolution in *Cereus* lineages. The workflow summarizes the analytical framework used in this study.

**Figure S2**. Predicted structures of two representative orthogroups: (A) OG0028003 and (B) OG0030099. Representative structural models of orthogroups OG0028003 and OG0030099. Protein backbones are shown in grey, lineage-specific substitutions in blue, and entropy-defined sequence variability hotspots in red. In these representative orthogroups, most substitutions and hotspots occur in surface-accessible regions rather than in structurally conserved cores, illustrating the localized nature of evolutionary divergence in these proteins.
